# Commentary: Perception and Reality: Why a Wholly Empirical Paradigm is Needed to Understand Vision

**DOI:** 10.3389/fnsys.2016.00077

**Published:** 2016-09-21

**Authors:** Luis H. Favela

**Affiliations:** Department of Philosophy and Cognitive Sciences Program, University of Central FloridaOrlando, FL, USA

**Keywords:** affordances, ecological psychology, information, perceptual systems, retinal image, vision, visual perception

This commentary attempts to make two points: First, although Purves, Morgenstern, and Wojtach (PMW) are likely correct that the retinal image alone is an insufficient basis for successful visually guided action (Purves et al., 2015a, p. 1), their “vision as reflexive” strategy emphasizes the perceptual associations and neural portions of the visual system at the cost of understating the central role environmental information plays in vision. Second, although PMW are correct that early empirical strategies did not sufficiently incorporate neural aspects of the visual system (pp. 2–4), recent attempts to demonstrate how affordances relate to the nervous system help demonstrate that ecological psychology (EP, Gibson, 1966/1983, 1979/1986) remains a viable empirical framework for investigating and explaining vision.

EP is in agreement with PMW that retinal images alone are insufficient for visually guided action (Purves et al., 2015a, p. 1). PMW's “vision as reflexive” strategy (Purves et al., 2015a) claims that the link between stimulus and behavior that gives rise to visual perceptions “does not entail representing reality as such” (Purves et al., 2015b, p. 2). What matters for vision is the strength of reflexive bonds underlying perceptual associations that are reinforced over evolutionary timescales (Purves et al., 2015a, pp. 6–8; Purves et al., 2015b, p. 2). Although there are parts of this “reflexive” strategy that are congruent with EP, the inferred, indirect, and representational conception of vision on offer by PMW is incompatible with EP's central theoretical commitments.

EP emphasizes the idea that vision occurs in *perceptual systems*, where “perceptual system” refers to the animal-environment system (Favela and Chemero, 2016). When visually-guided action occurs in animals like us, it does so with our eyes connected to a brain, in a head, on a neck, on a body, with legs that move in particular ways while engaging with an environment with particular features. Understood in this way, vision is just as much *action* as perception, and it is an action that unfolds over time. Though the retinal image alone may not be sufficient to specify properties of the environment such as size and distance, environmental information such as optic energy structures are rich enough. “Information” is used in EP in terms of energy distributions (e.g., light and sound) specific to the layout of the world, and is revealed to an animal via its perceptual capacities as it moves. Along these lines, information is a systems-based, relational feature between animal and environment (Chemero, 2009). Energy distributions surrounding an animal can be rich with information that specifies action-relevant properties of the world (Fajen et al., 2008). Such properties are *affordances*, or opportunities for action. The perception of affordances has been demonstrated in investigations of the perception of gap sizes (Barac-Cikoja and Turvey, 1993), stand-on-ability of slopes (Fitzpatrick et al., 1994), catching moving objects (Oudejans et al., 1996), sit-on-ability (Mark, 1987), step-on-ability (Warren, 1984), and the pass-through-ability of apertures (Warren and Whang, 1987), just to name a few. Appealing to the structure of environmental information when accounting for the results is common across all of these perceptual experiments.

Temporality plays another major role in the ability of an animal to perceive affordances. Consider that a tree's distance is not specified by the retinal image alone, i.e., a tree that is small and near would cast the same retinal image as one large and far. However, since animals are always in motion, environmental cues such as motion parallax can facilitate the specification of features of objects such as size and distance. These considerations lend support to the current claim that although PMW's “vision as reflexive” strategy is correct to shift focus away from the retinal image as the basis of vision, they need not understate the richness of information available in the environment to specify features relevant to visually-guided behavior. Furthermore, the sample of studies above lend support to the notion that, contra PMW, environmental information can specify an animal's reality as such.

Next, I draw attention to the continued viability of EP as an empirical framework for investigating and explaining vision. PMW state that, like other early empirical strategies, EP has, “suffered from an absence of ties to the structure and function of animal visual systems” (Purves et al., 2015a, p. 3). This is a justified criticism. Although ecological psychologists do not study the nervous system *per se* (e.g., single neuron recordings, etc.), investigations friendly to EP at the intersection of the neural sciences and perceptual psychology have begun to connect research on affordance perception and associated neural physiology. Directly inspired by Gibson, Cisek's “affordance competition hypothesis” (Cisek, 2007; Cisek and Kalaska, 2010) takes an embodied, sensorimotor approach to explaining the neural underpinnings of how animals make action selections amid environments containing multiple relevant affordances. Duran and Thill (2012) present the “dynamic field theory” as a neurophysiologically inspired dynamical systems framework for modeling relationships among affordances and language. Mizelle et al. (2013) investigated potential neural networks underlying the perception of affordances that could facilitate goal-directed actions (i.e., “functional affordance”) and those affordances that are actually used to achieve a goal (i.e., “physical affordance”). In addition, Young (2006) utilized evidence from patients with visual pathologies to develop a way to categorize affordances based upon their neurological underpinnings.

Though some of these studies modify Gibson's original conception of affordances (e.g., Gibson, 1979/1986), they remain compatible with the EP strategy as a whole, particularly the role affordances play in direct perception (cf. Michaels and Carello, 1981; de Wit et al., 2015). By incorporating relevant neurophysiological features of the visual system into accounts of affordance perception, EP remains a viable *empirical* framework for investigating and explaining vision. Moreover, it remains a framework that need not abandon its commitments to central tenets such as perceptual systems as the objects of investigation and environmental information as being rich enough to specify features of the world.

## Author contributions

The author confirms being the sole contributor of this work and approved it for publication.

### Conflict of interest statement

The author declares that the research was conducted in the absence of any commercial or financial relationships that could be construed as a potential conflict of interest.
